# The Advancement of Women

**Published:** 1893-04

**Authors:** 


### HALL'S JOURNAL OF HEALTH

TRUTH DEMANDS NO SACRIFICE; ERROR CAN MAKE NONE.

Vol. 40. APRIL, 1893. No. 4.

### THE ADVANCEMENT OF WOMEN.

We are in a drift toward the education of women such as has not been before known in this country, and the industrial openings to them are equal to the impetus of the desire for their increased education. Ten years ago no one would have dared to prophesy the character of the present movement toward their advanced education, and already the results of this wider training are beginning to be felt in a larger understanding of what women can do and a larger opening of usefulness to them in the active walks of life. We are rapidly disabusing society of the old idea that it is not the thing for a woman to have a purpose in life, and that she is doomed to allow herself to live like a plant in the shade or like a weed in the garden, not brought out into the light, not specially trained and prepared for the duties of life. The old idea has been to give a boy who had any promise a careful education, and to stir up his ambition at the point where the boy develops into the young man as much as possible, but at this point in the old education the severity of a girl's training was diminished. She not only was allowed freedom, and the real purpose of life seemed to be absent, but she was not expected to come to anything, and, therefore, she was not rigorously prepared for anything. At the present time this kind of training for young women has practically passed away. It is believed that every girl should be well educated, and the stress is applied to girls quite as much as to boys to make the most of themselves. You see this in the grammar schools, in the high schools, in the number of young women who are going to college, and in the very ordering of home life. Once a man who had half a dozen daughters was pitied because he had nobody to help him. The girls were good for nothing until they got married, and then they were not worth much, because they had never been properly trained, but under the lead of ideas which prevail to-day a family has a chance to make its mark through its girls perhaps quite as much as through the boys.

We have come to the time when a young woman has applied to her life all the spur which used to be applied exclusively to the lives of her brothers. She has a career before her, and it is not entirely the career of a married woman. So thoroughly are the young women who are to be the social and industrial leaders of the next generation imbued with this idea of purpose and career, and so much of it is a part of the spirit of our own time, that it is difficult to think of the future of women in almost any sphere of life who are not cultivated, or who are simply dissatisfied and unoccupied persons. It is to the credit of the higher education for women that it has broken up the old listlessness, and begun to make it fashionable for women to faithfully develop their mental gifts and use them for profit or for the benefit of others, as the duties of life may open out to them. No limit is placed to-day upon the attainments of women. If a woman has any qualities that make her a desirable member of society, she has a demand for their use, and it is this recognition of the superior mental service which women bring into life that has been the distinguishing feature of the advanced schools for women. They have simply widened out the range of woman's life until it has practically become analogous to that of men, not the same life, but a life that answers the same end.

We are likely to hear much less about the waste of woman's intellectual force in the future than we have in the past. The effect of this intellectual training upon women is to make them more desirable companions for men, their intellectual equals, their sympathetic appreciators, and to give women in their homes the opportunity to realize what may be accomplished in the training of their sons and daughters, and in the sending out of a strong influence into society. There are hundreds of topics on which women are soon to be able to converse with as much intelligence and freedom as men bring to them, and it is their destiny to take into the home, to a great extent, a newer and a higher and a more inspiring life. No one who has watched the gradual influence of the higher education in our social and domestic fields will deny that this is a noble feature of our social development.

Not only the multiplication of colleges for women, but the introduction of co-education in many of our leading universities is to do much toward the hastening of this change. The typical woman will in due time, be one who is educated all around, who has acquired true intellectual discipline and has learned how to apply a purpose to that discipline which stimulates and inspires the whole work of her life. The outlook for the next generation is bright and instructive. Who would not like to be a son or daughter of the mothers who are yet to be? Not that the mothers of the race of men and women of our time did not discharge their duty, but the educated mother of the future will be a woman who not only has capacity to bring life into the world, but the ability to guide it to the accomplishment of greater things than were possible in the old days. It is impossible to look forward without the increasing conviction that the larger culture which women are bringing to the work of life is to have a very important bearing upon the whole range of human activity, and the new race of men and women will begin where many of us have had to leave off.

